# Association of pre-existing cardiovascular disease with administration of fluoropyrimidine chemotherapy in patients with gastrointestinal malignancies

**DOI:** 10.1136/bmjonc-2024-000323

**Published:** 2024-08-08

**Authors:** Aderonke Temilade Abiodun, Chengsheng Ju, Catherine A Welch, Jennifer Lai, Freya Tyrer, Pinkie Chambers, Lizz Paley, Sally Vernon, John Deanfield, Mark de Belder, Mark Rutherford, Paul C Lambert, Sarah Slater, Kai Keen Shiu, Li Wei, Michael D Peake, The VICORI collaborative, David Adlam, Charlotte Manisty

**Affiliations:** 1 University College London Institute of Cardiovascular Science, London, UK; 2 Barts Heart Centre, Barts Health NHS Trust, London, UK; 3 National Disease Registration Service, NHS England, Leeds, UK; 4 Research Department of Practice and Policy, School of Pharmacy, University College London, London, UK; 5 Department of Population Health Sciences, University of Leicester, Leicester, UK; 6 Cancer Division, University College London Hospitals NHS Foundation Trust, London, UK; 7 National Institute of Cardiovascular Outcomes Research (NICOR), NHS Arden and Greater East Midlands Commissioning Support Unit, Leicester, UK; 8 Department of Medical Epidemiology and Biostatistics, Karolinska Institutet, Stockholm, Sweden; 9 Barts Cancer Centre, Barts Health NHS Trust, London, UK; 10 Cancer Institute, University College London, London, UK; 11 Department of Respiratory Medicine, University of Leicester, Glenfield Hospital, Leicester, UK; 12 Cancer Research UK, Oxford, UK; 13 Department of Cardiovascular Sciences and NIHR Leicester Biomedical Research Centre, University of Leicester, Glenfield Hospital, Leicester, UK

**Keywords:** Chemotherapy, Colorectal cancer, Gastric cancer, Oesophageal cancer

## Abstract

**Objective:**

Fluoropyrimidine chemotherapy is a first-line treatment for many gastrointestinal (GI) cancers, however, cardiotoxicity concerns may limit administration in patients with pre-existing cardiovascular disease (CVD). This study investigated the association of pre-existing CVD with use of fluoropyrimidine chemotherapy in tumour-eligible GI cancer patients.

**Methods and analysis:**

National cancer registry data from the Virtual Cardio-Oncology Research Initiative from England between 2014 and 2018 was used to identify GI cancer patients eligible to receive fluoropyrimidine chemotherapy. Linkage to Hospital Episode Statistics and CVD registry data were used to ascertain prior CVD and outcomes. Primary outcome was first administration of fluoropyrimidine chemotherapy following cancer diagnosis. Cox proportional hazard models determined HR and 95% CIs for the association between initiation of fluoropyrimidine treatment and prior CVD.

**Results:**

112 726 eligible patients were identified (median age 71 years (IQR 62–80), 39.7% female). 33 026 (29.3%) had pre-existing CVD. 73 392 (65.1%) patients had a diagnosis of colorectal, 23 208 (20.6%) oesophageal, 14 788 (13.1%) gastric and 1338 (1.2%) small bowel cancer. Individuals with pre-existing CVD had a 27% reduced rate of receiving fluoropyrimidine chemotherapy (HR, 0.73; 95% CI 0.70 to 0.75) on multivariable analysis. Significantly reduced rates of fluoropyrimidine administration were found across all subtypes of pre-existing CVD.

**Conclusions:**

GI cancer patients with all types of pre-existing CVD are less likely to receive fluoropyrimidine chemotherapy despite eligibility. This suggests widespread caution regarding administration of fluoropyrimidines across this population; further research is needed to assess whether such conservatism is justified.

**WHAT IS ALREADY KNOWN ON THIS TOPIC**

- Fluoropyrimidine chemotherapy is used first-line in the treatment of gastrointestinal (GI) cancers, with limited alternative treatment options available. Fluoropyrimidine associated cardiotoxicity is an important potential complication and the relationship with pre-existing cardiovascular disease is unclear.

**WHAT THIS STUDY ADDS**

- This study highlights that, across patients with GI cancers eligible for treatment fluoropyrimidine chemotherapy, those with pre-existing cardiovascular disease (CVD) have 27% lower rates of fluoropyrimidine administration. This is seen across all subtypes of CVD including arrhythmia, and despite rates of pre-existing CVD of almost 30% in this population.

**HOW THIS STUDY MIGHT AFFECT RESEARCH, PRACTICE OR POLICY**

- Our findings highlight the importance of further research to determine the baseline risk predictors of fluoropyrimidine-associated cardiotoxicity to promote informed decision-making and avoid unnecessary therapeutic conservatism.

## INTRODUCTION

Cancers of the gastrointestinal (GI) tract constitute 26% of all new cancer diagnoses and are responsible for 35% of cancer-related deaths worldwide.^1^ Global figures for new cases and fatalities resulting from GI cancers are forecast to rise by 58% and 73%, respectively; reaching 7.5 million and 5.6 million by the year 2040.^1^ Fluoropyrimidines, 5-fluorouracil (5-FU) and its oral prodrug capecitabine, are antimetabolite chemotherapeutic agents that are the most common class of drugs used for treatment of GI malignancies, alongside other solid tumours. They form a core component of guideline-recommended treatment regimens for oesophageal and gastric cancers in the neoadjuvant, adjuvant and palliative settings, as well as the adjuvant and palliative settings for colorectal cancers.^2–9^

Fluoropyrimidines can lead to a range of systemic toxicities, one of the most important is cardiotoxicity. There is a variety of presentations of fluoropyrimidine-associated cardiotoxicity (FAC)—most commonly coronary spasm causing chest pain, but this can lead to an acute coronary syndrome and (rarely) sudden cardiac death. Heart failure, myocarditis and arrhythmias have also been described in association with fluoropyrimidines although the mechanisms are unclear.^10 11^ FAC is the second most common cause of chemotherapy-related cardiovascular toxicity, with the accepted incidence rate in most reviews considered to be between 1% and 19%.^11 12^ In studies assessing the incidence of FAC specifically in the GI cancer setting, the reported incidence ranges between 3% and 29.5%.^11^ The relationship between pre-existing cardiovascular disease (CVD) or cardiovascular risk factors and risk of FAC however remains unclear, with conflicting results from previous studies.^11 13–17^ Despite this lack of clear evidence, cancer clinicians are likely to exercise caution in prescribing fluoropyrimidines to patients with a significant cardiac history due to concerns regarding potential cardiotoxicity. Given the lack of available effective second-line non-fluoropyrimidine-containing chemotherapy regimens,^18–20^ cancer outcomes may be inferior in patients not deemed fit for fluoropyrimidine chemotherapy due to the perceived risk of cardiotoxicity. The European Society of Cardiology guidelines in cardio-oncology advise clinicians to perform a detailed general baseline cardiovascular risk assessment and to manage modifiable cardiovascular risk factors before commencing fluoropyrimidine treatment. The document also advises clinicians to consider screening for coronary artery disease (CAD) in patients at high risk. There is however no specific advice regarding which patients should not receive treatment from a cardiovascular perspective or provide specific cardiotoxicity risk stratification tools unlike for many other systemic anticancer therapies.^21^

In this study, we sought to study the impact of CVD on the prescription of fluoropyrimidine chemotherapy to patients with GI malignancies with a disease stage eligible to receive treatment across the whole of England, UK.

## Materials and methods

### Data source

We used de-identified data provided by the Virtual Cardio-Oncology Research Initiative (VICORI) programme for this study.^22^ VICORI is a research platform that links the National Cancer Registration Dataset (NCRD),^23^ National Institute for Cardiovascular Outcomes Research (NICOR) data for CVD registries, Hospital Episode Statistics (HES) for hospital admission data and Office for National Statistics death registry data through a unique identifier.^24^ In this study, four NICOR databases were included: the Myocardial Ischemia National Audit Project (MINAP),^25^ the National Audit Cardiac Surgery Audit (NACSA),^26^ the National Audit Percutaneous Coronary Intervention (NAPCI)^27^ and the National Heart Failure Audit (NHFA).^28^ The NCRD was also linked with the Systemic Anti-Cancer Therapy (SACT) database for the identification of systemic anticancer therapies for the patient population, with data submitted by individual NHS Trusts.^29^ The data quality for SACT was deemed to be sufficient for the proposed analysis for diagnoses from 2014 onwards.^29^ To ensure the availability of complete cardiovascular datasets, a 10-year lookback window was selected.

### Study design and study population

This was an observational cohort study including patients aged between 18 and 100 years in England with a first diagnosis of GI cancer (International Classification of Diseases (ICD)-10 codes C15-C21)^30^ between 1 January 2014 and 31 March 2018, who were identified from the NCRD data. We included patients with recorded age, gender, stage II–IV oesophageal and gastric cancers, and stage III–IV small bowel and colorectal cancers, for which neoadjuvant/adjuvant or palliative fluoropyrimidine is indicated.^5 6^ We excluded patients with tumour histology types for which fluoropyrimidines are not indicated (melanoma, sarcoma, carcinoid, neuroendocrine, mesenchymal and Paget’s disease). The stage of the tumour was calculated based on the tumour, node, metastases staging. Patients with missing vital status or no follow-up, and those without linkage with HES data, or cancer stage (non-inferable) were further excluded. All patients were followed from the cancer diagnosis until the study outcome, death or 1 year after the cancer diagnosis, whichever occurred first.^24^

### Exposure

The exposures of the study were pre-existing CVDs within 10 years prior to cancer diagnosis, which included CAD (angina pectoris, acute coronary syndrome, percutaneous coronary intervention and coronary artery bypass grafting), deep venous thromboembolism, pulmonary embolism, heart failure and cardiomyopathy, valvular heart diseases, arrhythmia, peripheral vascular disease (PVD), sudden cardiac arrest and stroke. The pre-existing CVD status was assessed from MINAP, NACSA, NAPCI, NHFA and HES data. The definition of each of the study variables can be found in online supplemental table S1.

10.1136/bmjonc-2024-000323.supp1 Supplementary data

### Outcome

The primary outcome was the first prescription of fluoropyrimidine chemotherapy after cancer diagnosis. The fluoropyrimidine treatment included 5-FU or capecitabine monotherapy or in combination with other drugs.

### Covariates

Covariates that have been included in the adjustment were: age at cancer diagnosis, gender, ethnicity group (White, Asian, Black, Mixed and Other), Index of Multiple Deprivation (IMD) 2019 quintiles for socioeconomic status,^31^ tumour site (based on ICD-10 codes), tumour stage (American Joint Committee of Cancer), route to diagnosis of cancer^32^ and previous hospitalisation for hypertension, type II diabetes mellitus and chronic kidney disease. Tumour histology and performance status (PS) were described as additional baseline characteristics.

The covariates integrated for adjustment were carefully chosen based on clinical knowledge, indicating their expected association with baseline CVD and/or fluoropyrimidine treatment.

### Statistical analysis

Baseline characteristics were presented as median (IQR) for continuous variables and as numbers (%) for categorical variables. Standardised mean difference (SMD) was used to evaluate the differences in baseline variables between groups. An SMD greater than 0.1 was considered as significant difference between groups.

Time-to-event analyses with Cox proportional hazard models were used to estimate the comparative rate of fluoropyrimidine treatment by exposure category, with the reference category being patients with no pre-existing CVD. The choice of Cox model was based on the hypothesis that pre-existing CVD may lead to avoidance of or delayed fluoropyrimidine treatment. The proportionality assumption for the Cox model was checked by visually inspecting the slope of Schoenfeld residuals prior to statistical testing. The Cox proportional hazards models were used to calculate HRs and 95% CIs. Then multivariable Cox models were used to adjust for the effects of measured covariates on the receipt of fluoropyrimidine treatment. Age was included as a linear term. Death was considered as a potential competing risk event and as a censoring event in cause-specific analyses. We also conducted additional analysis on death prior to fluoropyrimidine treatment as an additional study outcome using the Cox proportional hazard models with the same covariates plus CVD. Standardised cumulative incidence plots were also plotted to assess the absolute probabilities of receiving fluoropyrimidine or death without fluoropyrimidine treatment over the follow-up period. All the standardised cumulative incidence plots were adjusted by the measured baseline covariates.

A two-sided p value <0.05 was considered statistically significant. All statistical analyses were performed with R V.4.3.1. The standardised cumulative incidence plots were calculated using ‘adjustedcif’ package with direct standardisation.

### Subgroup analysis and sensitivity analysis

We conducted two prespecified subgroup analyses: (1) analysis stratified by individual CVD category exposures; (2) analysis stratified by tumour site, based on the ICD-10 codes (oesophageal cancer (C15), gastric cancer (C16), small intestinal cancer (C17) and colorectal cancer (C18-21)).

Further sensitivity analyses included (1) extending follow-up of all patients until the October 2022 data cut, rather than 1 year after the cancer diagnosis; (2) inclusion of the diagnosis hospital as an additional covariate in the Cox model; (3) including PS in the model, with patients with missing PS or PS=4 excluded; (4) including PS in the model, with patients with missing PS or PS=3 or 4 excluded. Patients with a PS of 3 or 4 were excluded due to the low likelihood of receiving any systemic chemotherapy^33 34^; (5) conducting a logistic regression to investigate the probability of receiving fluoropyrimidine treatment within 1 year associated with baseline CVD status, rather than a Cox regression so to ignore the difference in timing of treatment.

## Results

### Study cohort and baseline demographics

**Figure 1:**
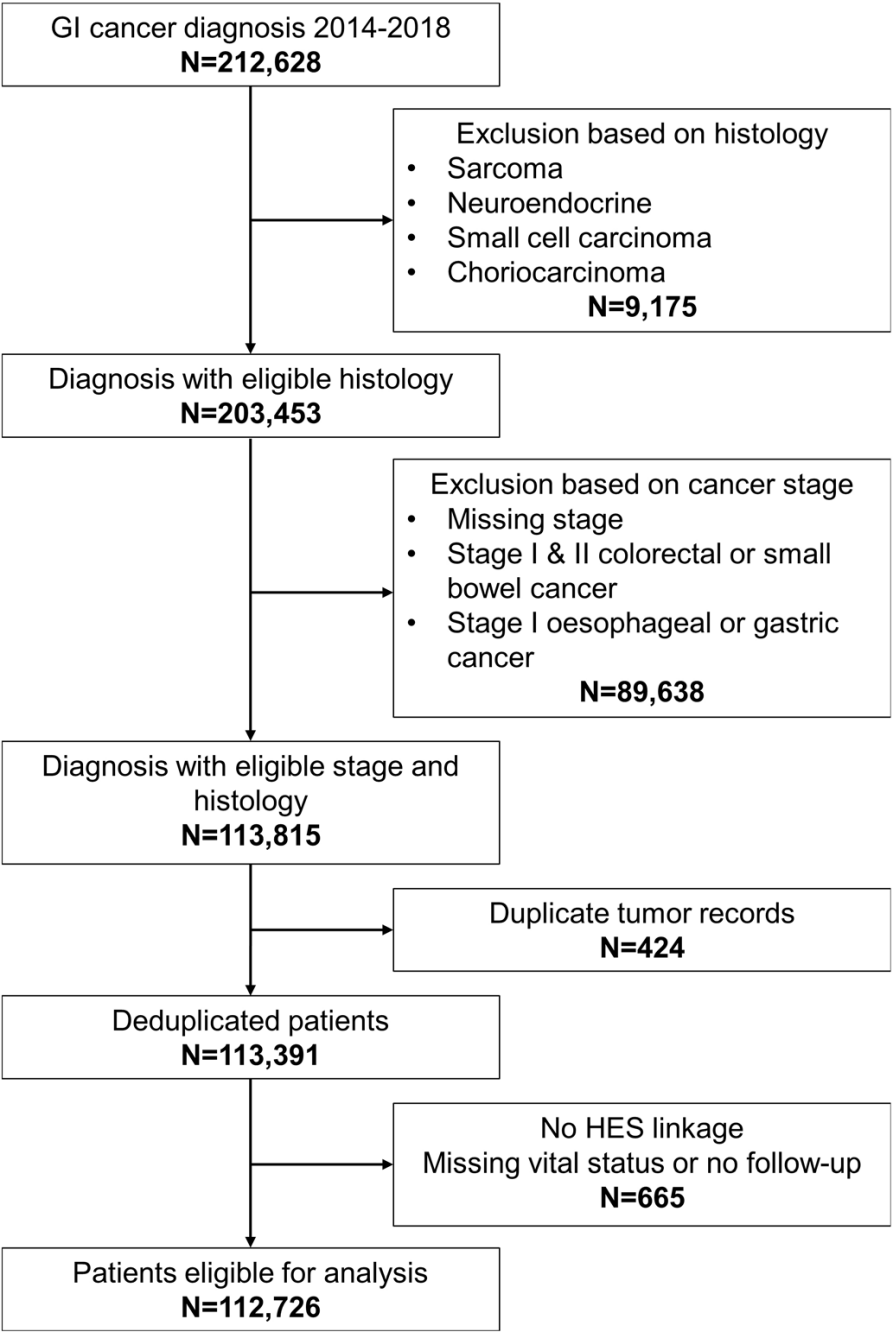
Patient selection process. GI, gastrointestinal; HES, Hospital Episode Statistics.

We extracted data from 212 628 cancer diagnoses in England between 1 January 2014 and 31 March 2018. Patients were excluded where there were missing or ineligible data for tumour stage (n=89 638), tumour cell histology (n=9175), where subsequent tumour diagnoses were made after an initial diagnosis (n=424), where linked HES records were unavailable (n=607) or where vital status was missing or there was no follow-up (n=58). After exclusions, 112 726 patient records were included in the final analysis (figure 1). Median age was 71 years (IQR, 62–80), and 44 759 (39.7%) of patients were female. 102 948 (91.3%) of patients were of white ethnicity and 21 004 (18.6%) had the IMD score 1 (most deprived) (table 1).

**Table 1:** Baseline patient characteristics of all eligible patients and stratified by fluoropyrimidine treatment

	All patients N=112 726	Fluoropyrimidine N=57 957	No fluoropyrimidine N=54 769	SMD
Age in years (median (IQR))	71 (62, 80)	66 (58, 73)	78 (70, 84)	−1.02
Female (%)	44 759 (39.7)	21 682 (37.4)	23 077 (42.1)	0.10
Ethnicity (%)				0.10
White	102 948 (91.3)	53 014 (91.5)	49 934 (91.2)	
Asian	2408 (2.1)	1364 (2.4)	1044 (1.9)	
Black	1831 (1.6)	1017 (1.8)	814 (1.5)	
Mixed	410 (0.4)	232 (0.4)	178 (0.3)	
Other	1165 (1.0)	709 (1.2)	456 (0.8)	
Unknown	3964 (3.5)	1621 (2.8)	2343 (4.3)	
Index of Multiple Deprivation quintile (%)				0.08
1—most deprived	21 004 (18.6)	9961 (17.2)	11 043 (20.2)	
2	21 781 (19.3)	11 090 (19.1)	10 691 (19.5)	
3	23 244 (20.6)	12 128 (20.9)	11 116 (20.3)	
4	23 822 (21.1)	12 603 (21.8)	11 219 (20.5)	
5—least deprived	22 875 (20.3)	12 175 (21.0)	10 700 (19.5)	
Performance status (%)				0.81
0	29 661 (26.3)	22 449 (38.7)	7212 (13.2)	
1	21 042 (18.7)	12 355 (21.3)	8687 (15.9)	
2	9636 (8.6)	2705 (4.7)	6931 (12.7)	
3	5676 (5.0)	452 (0.8)	5224 (9.5)	
4	1111 (1.0)	44 (0.1)	1067 (2.0)	
Unknown	45 600 (40.5)	19 952 (34.4)	25 648 (46.8)	
Cancer site (%)				0.16
Oesophageal	23 208 (20.6)	11 784 (20.3)	11 424 (20.9)	
Gastric	14 788 (13.1)	6244 (10.8)	8544 (15.6)	
Small intestine	1338 (1.2)	556 (1.0)	782 (1.4)	
Colorectal	73 392 (65.1)	39 373 (67.9)	34 019 (62.1)	
Cancer histology (%)				0.59
Adenocarcinoma	91 213 (80.9)	51 600 (89.0)	39 613 (72.3)	
Squamous cell carcinoma	9391 (8.3)	5126 (8.8)	4265 (7.8)	
Other	12 122 (10.8)	1231 (2.1)	10 891 (19.9)	
Cancer stage (%)				0.27
II	6996 (6.2)	3484 (6.0)	3512 (6.4)	
III	54 125 (48.0)	31 491 (54.3)	22 634 (41.3)	
IV	51 605 (45.8)	22 982 (39.7)	28 623 (52.3)	
Route to diagnosis (%)				0.55
Emergency presentation	27 626 (24.5)	8579 (14.8)	19 047 (34.8)	
GP referral	22 283 (19.8)	11 358 (19.6)	10 925 (20.0)	
Inpatient elective	5278 (4.7)	3139 (5.4)	2139 (3.9)	
Other outpatient	6275 (5.6)	3136 (5.4)	3139 (5.7)	
Screening	5099 (4.5)	4072 (7.0)	1027 (1.9)	
Two-week wait	44 199 (39.2)	26 846 (46.3)	17 353 (31.7)	
Unknown	1966 (1.7)	827 (1.4)	1139 (2.0)	
Previous hospitalisation (%)				
Arrhythmia	13 731 (12.2)	3353 (5.8)	10 378 (19.0)	−0.41
Previous cardiac arrest	493 (0.4)	69 (0.1)	424 (0.8)	−0.10
Chronic kidney disease	3317 (2.9)	435 (0.8)	2882 (5.3)	−0.26
Coronary artery disease	17 886 (15.9)	5602 (9.7)	12 284 (22.4)	−0.35
Diabetes mellitus	7727 (6.9)	2033 (3.5)	5694 (10.4)	−0.27
Heart failure	6374 (5.7)	1131 (2.0)	5243 (9.6)	−0.33
Hypertension	22 293 (19.8)	6280 (10.8)	16 013 (29.2)	−0.47
Peripheral vascular disease	2746 (2.4)	750 (1.3)	1996 (3.6)	−0.15
Stroke	3379 (3.0)	692 (1.2)	2687 (4.9)	−0.22
Valvular heart disease	5108 (4.5)	1186 (2.1)	3922 (7.2)	−0.25
Venous thromboembolic disease	3981 (3.5)	1090 (1.9)	2891 (5.3)	−0.18
Any cardiovascular disease*	33 026 (29.3)	9948 (17.2)	23 078 (42.1)	−0.57

*Any of the following diagnoses: angina, myocardial infarction, revascularisation procedures, percutaneous coronary interventions, coronary artery bypass graft surgery, deep venous thromboembolism, pulmonary embolism, heart failure and cardiomyopathy, valvular heart diseases, arrhythmia, peripheral vascular disease, sudden cardiac arrest and stroke.

GP, general practitioner; SMD, standardised mean difference.

73 392 (65.1%) of patients had a diagnosis of colorectal cancer, 23 208 (20.6%) had oesophageal cancer, 14 788 (13.1%) had gastric cancer and 1338 (1.2%) had cancer of the small intestine. 6996 (6.2%) had stage II disease at presentation, 54 125 (48.0%) had stage III disease and 51 605 (45.8%) had stage IV disease (table 1). The majority of patients 44 199 (39.2%) were diagnosed via the 2-week wait referral pathway,^35^ 27 626 (24.5%) were diagnosed during an emergency admission and 5099 (4.5%) of the cancer diagnoses were made via the National Bowel Cancer Screening Programme.

33 026 (29.3%) of patients had pre-existing CVD at the time of their cancer diagnosis. 13 731 (12.2%) had arrhythmia, 17 886 (15.9%) CAD, 493 (0.4%) previous cardiac arrest, 6374 (5.7%) heart failure, 2746 (2.4%) PVD, 3379 (3.0%) stroke, 5108 (4.5%) valvular heart disease and 3981 (3.5%) had venous thromboembolic disease. The baseline patient characteristics stratified by CVD status were presented in online supplemental table S2.

Overall, 57 957 (51.3%) of patients received fluoropyrimidine chemotherapy within 1 year of cancer diagnosis. Patients who received fluoropyrimidines exhibited significant differences, being younger, lower PS, having fewer comorbidities, and presenting with earlier disease onset, and differences in the presentation of route to diagnosis, tumour stage and histology compared with patients who did not receive fluoropyrimidines (table 1). The descriptions of the patient characteristics in patients with baseline CVD stratified by fluoropyrimidine treatment were in online supplemental table S3. Similar patterns were observed as in the total study cohorts.

### Fluoropyrimidine chemotherapy prescription and association with prior CVD

9948 (17.2%) of patients receiving fluoropyrimidines had pre-existing CVD at baseline, compared with 23 078 (42.1%) of patients not receiving fluoropyrimidines (table 1).

**Figure 2:**
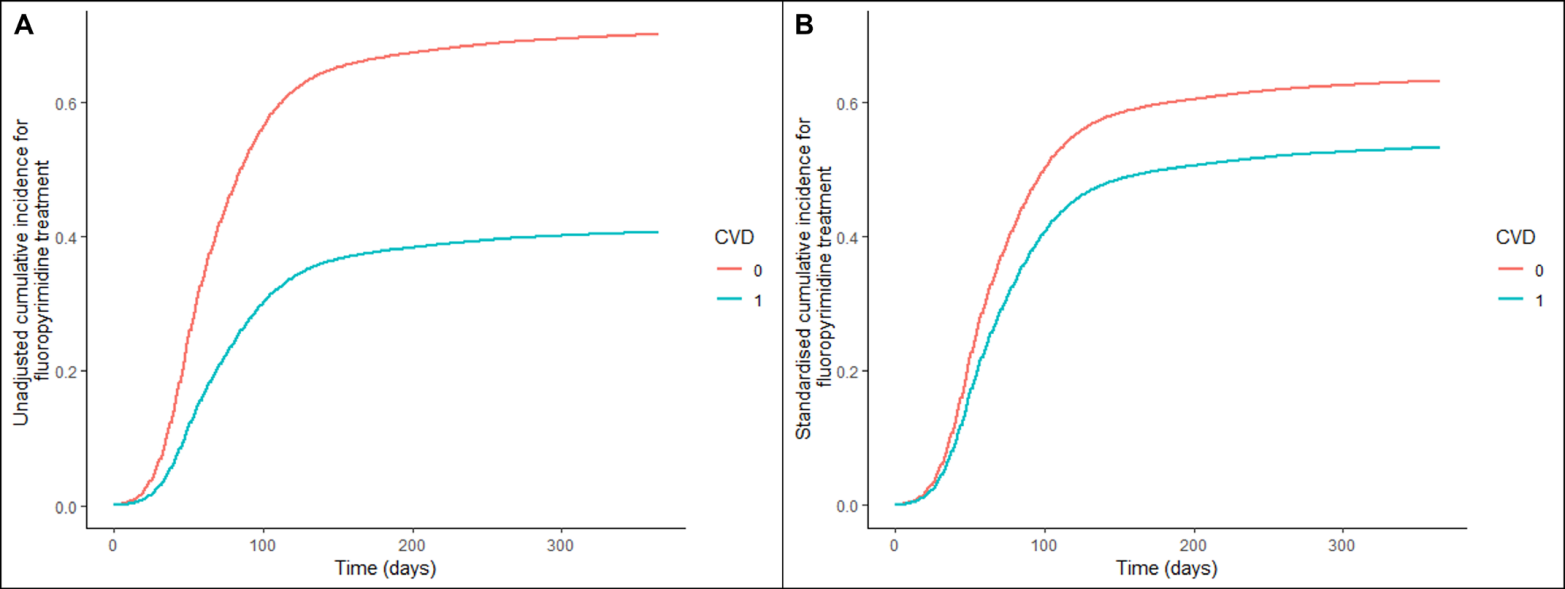
Cumulative incidence, stratified by pre-existing cardiovascular disease (CVD) status of receiving fluoropyrimidine chemotherapy, (A) unadjusted; (B) standardised.

Patients with CVD had a 57% lower rate of receiving fluoropyrimidine treatment than those without CVD (HR, 0.43; 95% CI 0.42 to 0.44). This remained significant after accounting for baseline covariates on multivariable analysis (HR, 0.73; 95% CI 0.70 to 0.75) (table 2). We also assessed the standardised cumulative incidence of receiving fluoropyrimidine chemotherapy over a 1-year period following diagnosis and compared this in patients with pre-existing CVD versus those who did not (figure 2). In patients with CVD and receiving treatment, the median time from diagnosis to commencing treatment was 63 days (IQR, 44–93), and 59 days (IQR, 42–85) in those without CVD. The specifications of the Cox model is presented in online supplemental table S4.

**Table 2:** Association of pre-existing cardiovascular disease with the rate of receipt of fluoropyrimidine treatment

	Event (n)	Total follow-up time (patient-years)*	Crude HR (95% CI)	Adjusted HR* (95% CI)
Fluoropyrimidine treatment				
CVD	9948	14 037	0.43 (0.42 to 0.44)	0.73 (0.70 to 0.75)
No CVD	48 009	26 970	Reference	Reference

*Adjusted for age at cancer diagnosis, gender, ethnicity, Index of Multiple Deprivation quintiles, tumour site, tumour stage, route to diagnosis of cancer, previous hospitalisation for hypertension, type II diabetes mellitus and chronic kidney disease.

CVD, cardiovascular disease.

When considering death as a competing risk, of patients who died without receiving fluoropyrimidine chemotherapy, 15 703 (44%) patients had CVD. In patients with CVD at baseline, there was a 57% increased rate of death prior to receipt of fluoropyrimidine chemotherapy compared with patients without CVD (HR, 1.57; 95% CI 1.54 to 1.60); 21% after multivariable adjustment (HR, 1.21; 95% CI 1.17 to 1.25) (online supplemental table S5).

### Effect of individual cardiovascular diagnoses on rate of prescription of fluoropyrimidine chemotherapy

**Figure 3:**
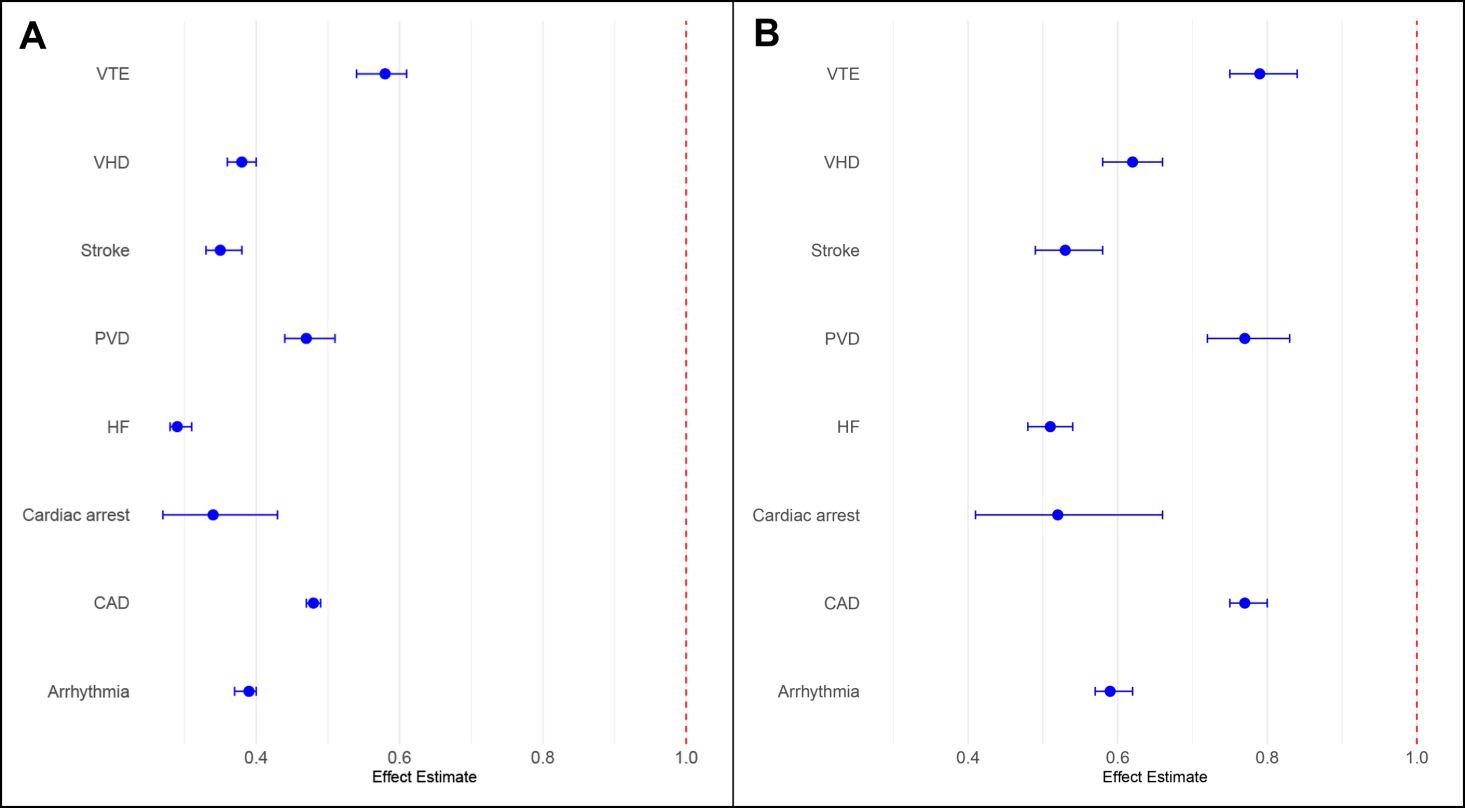
Forest plot of individual HRs of cardiovascular subtypes, (A) unadjusted; (B) adjusted*. *The estimates for each subtype were adjusted for age at cancer diagnosis, gender, ethnicity, Index of Multiple Deprivation quintiles, tumour site, tumour stage, route to diagnosis of cancer, previous hospitalisation for hypertension, type II diabetes mellitus and chronic kidney disease. CAD, coronary artery disease; HF, heart failure; PVD, peripheral vascular disease; VHD, valvular heart disease; VTE, venous thromboembolic disease.

Compared with patients without CVD, the rate of patients receiving fluoropyrimidines was lower for all individual CVD subtypes (figure 3); CAD HR, 0.77 (95% CI 0.75 to 0.80), heart failure HR, 0.51 (95% CI 0.48 to 0.54), prior stroke HR, 0.53 (95% CI 0.49 to 0.58), arrhythmia HR, 0.59 (95% CI 0.57 to 0.62), valvular heart disease HR, 0.62 (95% CI 0.58 to 0.66), prior venous thromboembolism HR, 0.79 (95% CI 0.75 to 0.84) and prior cardiac arrest HR, 0.52 (95% CI 0.41 to 0.66).

### Subgroup and sensitivity analyses

Irrespective of tumour location, patients with CVD had a lower rate of receiving fluoropyrimidine chemotherapy than those without CVD; oesophageal tumours HR, 0.76 (95% CI 0.71 to 0.81), gastric tumours HR, 0.66 (95% CI 0.60 to 0.73), small bowel tumours HR, 0.70, (95% CI 0.50 to 0.96), colorectal tumours HR, 0.73 (95% CI 0.70 to 0.76).

Results from sensitivity analyses were shown in online supplemental table S6. Adjusting for diagnosing hospital or extending the follow-up time in further sensitivity analyses had no significant effect on the results. PS is a strong predictor of whether a patient is likely to receive fluoropyrimidine treatment. After excluding 45 600 patients with missing PS and 1111 patients with a PS of 4, the HRs for PS 1, 2, 3 compared with 0 to receive fluoropyrimidines were 0.90 (95% CI 0.88 to 0.92), 0.46 (95% CI 0.44 to 0.48) and 0.16 (95% CI 0.15 to 0.18), respectively. After adjusting for PS, patients with CVD still had a reduced rate of receiving fluoropyrimidines (HR, 0.78; 95% CI 0.75 to 0.81). The baseline characteristics of the patients included in the analysis are presented in online supplemental table S7. When using a logistic regression model, baseline CVD status was associated with a reduced probability of receiving fluoropyrimidine treatment within 1 year (OR, 0.63; 95% CI 0.60 to 0.66).

## Discussion

In this study, we evaluated the association between pre-existing CVD and the likelihood of fluoropyrimidine chemotherapy prescription in patients with GI malignancies. Within our 112 726-patient cohort, more than one in four patients had prior CVD and these patients had a 27% lower rate of receiving fluoropyrimidine-based chemotherapy. A lower rate was observed in all specific subtypes of CVD.

Prior research examining the association between pre-existing CVD and cardiovascular risk factors with the risk of developing fluoropyrimidine-induced cardiotoxicity has yielded inconsistent findings. The most common cardiovascular event associated with fluoropyrimidine administration is coronary artery spasm, however, the evidence for this being more prevalent in patients with known CVD (even CAD) is weak. In a retrospective single-centre review of 452 female patients with breast cancer receiving capecitabine chemotherapy, patients with cardiac comorbidities were 5.5 times more likely to have cardiovascular events during treatment than those who did not.^13^ Similar results were seen in another retrospective analysis of 668 patients treated with fluoropyrimidines,^36^ and in a meta-analysis of 22 studies of approximately 21 000 patients,^11 16 37–46^ it was found that overall, patients with pre-existing cardiac disease had significantly higher risks of cardiovascular events during treatment than those without (pooled risk ratio of 3.26, 95% CI 2.15 to 4.95). In contrast, in a large single-centre retrospective analysis of 4019 patients with cancer receiving 5-FU where the clinical endpoint was limited to coronary vasospasm, it was found that patients with events during treatment were younger and less likely to have ischaemic heart disease or cardiovascular risk factors.^15^ It is therefore challenging to determine whether there is a causative effect of fluoropyrimidines on cardiovascular events in patients with pre-existing CVD. Current published evidence (largely retrospective) is also likely to be subject to ascertainment and publication bias, given the frequent exclusion of patients with known cardiac diseases from studies and clinical trials assessing fluoropyrimidine cardiotoxicity.^47–49^ If patients with pre-existing CVD are not at significantly increased risk of fluoropyrimidine cardiotoxicity, patients may in fact be receiving second-line cancer therapies inappropriately, and oncological outcomes may be compromised. This therefore requires further investigation. It is important to note that fluoropyrimidine metabolism is mediated predominantly by the enzyme dihydropyrimidine dehydrogenase (DPD) and due to the narrow therapeutic window, mutations in this enzyme have been associated with increased risks of systemic toxicities with this treatment.^50^ The relationship with DPD mutations and fluoropyrimidine associated cardiotoxicity is however unclear with some case reports suggesting an asscociation^51^ and other studies suggesting no significant association.^52 53^ DPD mutation status was not assessed routinely in clinical practice in the UK until within the past 5 years, and is not recorded in the National Cancer Datasets, therefore it was not feasible to determine its impact in this analysis.

Cancer and CVD have a number of shared risk factors and pathophysiological processes and hence they frequently coexist.^54^ More than a quarter of our patients with GI cancers had pre-existing CVD and this prevalence is higher than previously identified in a study of multiple cancers also using the VICORI dataset, where pre-existing CVD was found in 16.2% of all patients.^24^ We found that patients across all CVD subtypes were significantly less likely to be prescribed fluoropyrimidines, even for patients with conditions not known to be associated with fluoropyrimidine cardiotoxicity including valvular heart disease and venous thromboembolic diseases. It is possible that crude comorbidity criteria may be used in clinical decision-making, however, these differences in rates of prescription were independent of the overall well-being of the patients because the association persisted even after adjusting for the PS. The reduced likelihood of fluoropyrimidines being given to patients with prior CVD was consistent across all tumour types and therefore it is unlikely that availability of alternative regimens would explain the reason why fluoropyrimidines were not given.

To the best of our knowledge, this is the first study to explicitly explore the relationship between CVD and the administration of fluoropyrimidine chemotherapy, although prior studies have shown a similar effect with other chemotherapy agents. A prior retrospective population-based cohort study of 25 594 women with breast cancer showed that pre-existing CVD was associated with a lower likelihood of receiving any type of chemotherapy (OR 0.56),^55^ and a prospective study of 2127 women with newly diagnosed breast cancer also found lower rates of administration of adjuvant chemotherapy in patients with known CVD (OR 0.32).^56^

### Strengths and limitations

The study has several strengths. The VICORI initiative offers comprehensive detailed data linkage of the NCRDs and treatment datasets (SACT, HES), with CVD treatment registries for every hospital in England. The depth and breadth of data collection for cancer and CVD in VICORI helps to enable exposure and endpoint ascertainment.

There are however some limitations to our study. This is an observational cohort study and therefore, despite the strong inverse association found, we cannot conclude that the presence of pre-existing CVD is the cause of the reduction of exposure to fluoropyrimidines in this population. Our results do not have any causal interpretations because the exchangeability assumption between patients with and without pre-existing CVDs cannot be met, that is, patients with CVD are never comparable to patients without CVD. However, the intention of this study was not to make any causal inference but to test CVD as a risk factor for not receiving fluoropyrimidine treatment. Due to a high proportion of missing data, we were unable to include Eastern Co-Operative Group PS classification in our main analysis, which is known to be a significant determinant of patient suitability for chemotherapy used by oncologists.^29^ However, in the sensitivity analysis removing cases without PS, the association remained strong (5% absolute change in HR from 0.73 to 0.78). We did not have access to primary care records and were reliant on HES admission data and national cardiac registries for coding of CVD which has likely led to an underestimation of CVD prevalence by missing less severe CVDs without hospitalisation. The point estimate may be further attenuated if we adjust for residual confounding that may be available from other sources such as smoking history and body mass index. However, it is possible that hospitalised events may however have more impact on chemotherapy decision-making. A substantial proportion of patients were also excluded due to missing cancer stage and this may affect the generalisability of our findings. Finally, we could not rule out that patients with CVD are less likely to be treated with fluoropyrimidine because they had higher risk of death. We analysed death without fluoropyrimidine as a competing risk event and showed that the risk of death prior to fluoropyrimidine treatment was 21% higher in patients with baseline CVD after adjusting for covariates. However, we could not differentiate deaths as a true competing risk event or as the consequence of not being treated by fluoropyrimidines.

## Conclusions

Patients with pre-existing CVD are less likely to be administered first-line fluoropyrimidine chemotherapy with eligible GI cancers, irrespective of CVD type, and despite a lack of clear association of baseline cardiovascular risk with fluoropyrimidine cardiotoxicity. Continued research is needed to identify risk predictors, given that unwarranted therapeutic conservatism may compromise cancer outcomes.

## Data Availability

Data may be obtained from a third party and are not publicly available. Patient-level electronic health records obtained through VICORI can be obtained by successfully applying for access to linked VICORI data by contacting vicori@le.ac.uk. An application for data access is subject to approval of a project proposal, analysis plan and required data by the VICORI Project Review Panel. The authors will share programming code and aggregate statistics if requested.
