# Supplementary material for: Association of pre-existing cardiovascular disease with administration of fluoropyrimidine chemotherapy in patients with gastrointestinal malignancies
Source: BMJ Oncol. 2024 Aug 8;3(1):e000323. doi: 10.1136/bmjonc-2024-000323 (PMC11347681; doi:10.1136/bmjonc-2024-000323)
Supplement: Supplementary data [file bmjonc-2024-000323supp001.pdf]

Association of pre-existing cardiovascular disease with administration of fluoropyrimidine chemotherapy in patients with gastrointestinal malignancies

Supplementary materials

Supplementary table S1. Definition of study variables.

Variable	Definition
Age at cancer diagnosis	Provided by NCRD
Gender	Provided by NCRD, patient self-reported gender.
Ethnicity group	Provided by NCRD
IMD quintiles	Provided by NCRD
Performance status	Provided by NCRD
Tumour site	Provided by NCRD, ICD-10 codes C15-C21
Tumour stage	Provided by NCRD
Tumour grade	Provided by NCRD
Tumour histology	Provided by NCRD
Route to diagnosis of cancer	Provided by NCRD
CKD	HES, ICD-10 codes N18.3-N18.5, D63.1, E10.2, E11.2, E12.2, E13.2, E14.2, I12, I13, N03-N05, Z94.0
Hypertension	HES, ICD-10 codes I10
Type II diabetes mellitus	HES, ICD-10 codes E11
CAD	HES, ICD-10 codes I20-I25
	HES, OPCS-4 codes K40-K46
	MINAP, excluding diagnosis code 6, 8, 9 (unconfirmed diagnosis)
	HES, OPCS-4 codes K40-K46, K49, K50, K75
	NAPCI, any record
	NACSA, CABG=1
VHD	HES, ICD-10 codes I01-I08, I34-I37

	NACSA, VALVE=1
VTE	HES, ICD-10 codes I80.1-I80.3, I26
HF and cardiomyopathy	HES, ICD-10 codes I25.5, I42, I43, I50
	NHFA, any record
Cardiac arrhythmia	HES, ICD-10 codes I48, I49
Stroke	HES, ICD-10 codes I60-I64
PVD	HES, ICD-10 codes I73
Cardiac arrest	HES, ICD-10 codes I46
Fluoropyrimidine treatment	SACT, 5-FU or capecitabine analysis group
	AV treatment, 5-FU or capecitabine
Death	ONS death records

IMD=Index of Multiple deprivation, CKD=chronic kidney disease, CAD=coronary artery disease, HF=heart failure, PVD=peripheral vascular disease, VHD= valvular heart disease and VTE=venous thromboembolic disease.

Supplementary table S2. Baseline patient characteristics stratified by CVD status.

	**CVD**<br>**N=33,026**	**No CVD**<br>**N=79,700**
**Age in years (median [IQR])**	78 [70, 84]	69 [59, 77]
**Female (%)**	11,442 (34.6)	33,317 (41.8)
**Ethnicity (%)**		
White	31,086 (94.1)	71,862 (90.2)
Asian	565 (1.7)	1,843 (2.3)
Black	364 (1.1)	1,467 (1.8)
Mixed	88 (0.3)	322 (0.4)
Other	220 (0.7)	938 (1.2)
Unknown	703 (2.1)	3,268 (4.1)
**Index of Multiple deprivation quintile (%)**		
1 - most deprived	6,753 (20.4)	14,251 (17.9)
2	6,472 (19.6)	15,309 (19.2)
3	6,705 (20.3)	16,539 (20.8)
4	6,833 (20.7)	16,989 (21.3)
5 - least deprived	6,263 (19.0)	16,612 (20.8)
**Cancer site (%)**		
Oesophageal	7,007 (21.2)	16,201 (20.3)
Gastric	5,162 (15.6)	9,626 (12.1)
Small intestine	424 (1.3)	914 (1.1)
Colorectal	20,433 (61.9)	52,959 (66.4)
**Cancer histology (%)**		
Adenocarcinoma	24,928 (75.5)	66,285 (83.2)
Squamous cell carcinoma	2,473 (7.5)	6,918 (8.7)
Other	5,625 (17.0)	6,497 (8.2)

**Cancer stage (%)**		
II	2,459 (7.4)	4,537 (5.7)
III	14,898 (45.1)	39,227 (49.2)
IV	15,669 (47.4)	35,936 (45.1)
**Route to diagnosis (%)**		
Emergency presentation	11,798 (35.7)	15,828 (19.9)
GP referral	6,529 (19.8)	15,754 (19.8)
Inpatient elective	1,044 (3.2)	4,234 (5.3)
Other outpatient	2,173 (6.6)	4,102 (5.1)
Screening	845 (2.6)	4,254 (5.3)
Two-week wait	10,374 (31.4)	33,825 (42.4)
Unknown	263 (0.8)	1,703 (2.1)
**Previous hospitalization (%)**		
Chronic kidney disease	3,290 (10.0)	27 (0.0)
Diabetes mellitus	7,572 (22.9)	155 (0.2)
Hypertension	21,842 (66.1)	451 (0.6)

IQR=interquartile range.

Supplementary table S3. Baseline patient characteristics of patients with baseline cardiovascular disease stratified by fluoropyrimidine treatment.

	**Fluoropyrimidine N=9,948**	**No fluoropyrimidine N=23,078**
**Age in years (median [IQR])**	71 [65, 76]	81 [74, 86]
**Female (%)**	2,651 (26.7)	8,791 (38.1)
**Ethnicity (%)**		
White	9,438 (94.9)	21,648 (93.8)
Asian	188 (1.9)	377 (1.6)
Black	112 (1.1)	252 (1.1)
Mixed	23 (0.2)	65 (0.3)
Other	66 (0.7)	154 (0.7)
Unknown	121 (1.2)	582 (2.5)
**Index of Multiple deprivation quintile (%)**		
1 - most deprived	1,832 (18.4)	4,921 (21.3)
2	1,924 (19.3)	4,548 (19.7)
3	2,049 (20.6)	4,656 (20.2)
4	2,156 (21.7)	4,677 (20.3)
5 - least deprived	1,987 (20.0)	4,276 (18.5)
**Performance status (%)**		
0	2,914 (29.3)	2,100 (9.1)
1	2,685 (27.0)	3,483 (15.1)
2	791 (8.0)	3,297 (14.3)
3	143 (1.4)	2,744 (11.9)
4	9 (0.1)	571 (2.5)
Unknown	3,406 (34.2)	10,883 (47.2)
**Cancer site (%)**		

Oesophageal	2,276 (22.9)	4,731 (20.5)
Gastric	1,282 (12.9)	3,880 (16.8)
Small intestine	103 (1.0)	321 (1.4)
Colorectal	6,287 (63.2)	14,146 (61.3)
**Cancer histology (%)**		
Adenocarcinoma	8,854 (89.0)	16,074 (69.7)
Squamous cell carcinoma	853 (8.6)	1,620 (7.0)
Other	241 (2.4)	5,384 (23.3)
**Cancer stage (%)**		
II	760 (7.6)	1,699 (7.4)
III	5,163 (51.9)	9,735 (42.2)
IV	4,025 (40.5)	11,644 (50.5)
**Route to diagnosis (%)**		
Emergency presentation	1,849 (18.6)	9,949 (43.1)
GP referral	2,140 (21.5)	4,389 (19.0)
Inpatient elective	387 (3.9)	657 (2.9)
Other outpatient	737 (7.4)	1,436 (6.2)
Screening	569 (5.7)	276 (1.2)
Two-week wait	4,210 (42.3)	6,164 (26.7)
Unknown	56 (0.6)	207 (0.9)
**Previous hospitalization (%)**		
Chronic kidney disease	427 (4.3)	2,863 (12.4)
Diabetes mellitus	1,967 (19.8)	5,605 (24.3)
Hypertension	6,058 (60.9)	15,784 (68.4)

IQR=interquartile range.

Supplementary table S4. Multivariable analysis on the association between each covariate and the rate of receipt of fluoropyrimidines.

**Covariate**	**HR (95% CI)**
**Age**	0.96 (0.96-0.96)
**Gender**	
Male	Reference
Female	0.90 (0.88-0.91)
**Ethnicity**	
Asian	Reference
White	1.25 (1.18-1.32)
Black	1.11 (1.03-1.21)
Mixed	1.11 (0.96-1.27)
Other	1.11 (1.02-1.22)
Unknown	0.97 (0.90-1.04)
**Index of Multiple deprivation**	
1 - most deprived	Reference
2	1.10 (1.07-1.12)
3	1.19 (1.15-1.22)
4	1.21 (1.18-1.24)
5 - least deprived	1.22 (1.19-1.26)
**Cancer site**	
Oesophageal	Reference
Gastric	0.95 (0.92-0.98)
Small intestine	0.69 (0.63-0.75)
Colorectal	0.82 (0.80-0.84)
**Cancer stage (%)**	
II	Reference

III	1.01 (0.97-1.05)
IV	1.26 (1.21-1.31)
**Route to diagnosis**	
Emergency presentation	Reference
GP referral	1.07 (1.04-1.10)
Inpatient elective	1.12 (1.08-1.17)
Other outpatient	1.12 (1.08-1.17)
Screening	1.67 (1.61-1.74)
Two-week wait	1.64 (1.60-1.69)
Unknown	0.60 (0.56-0.65)
**Previous hospitalization**	
Hypertension	0.91 (0.87-0.95)
Diabetes mellitus	0.83 (0.79-0.88)
Chronic kidney disease	0.50 (0.46-0.55)
Cardiovascular disease	0.73 (0.70-0.75)

HR=hazard ratio, CI=confidence interval.

Supplementary table S5. Association of pre-existing cardiovascular disease with the rate of death without fluoropyrimidine treatment.

	**Event (n)**	**Total follow-up time (patient-years)***	**Crude HR (95% CI)**	**Adjusted HR* (95% CI)**
**Death without receiving fluoropyrimidine treatment**				
CVD	15,703	14,037	1.57 (1.54-1.60)	1.21 (1.17-1.25)
No CVD	19,784	26,970	Reference	Reference

CVD=cardiovascular disease, HR=hazard ratio, CI=confidence interval.

*Adjusted for age at cancer diagnosis, gender, ethnicity, Index of Multiple Deprivation (IMD) quintiles, tumour site, tumour stage, route to diagnosis of cancer, previous hospitalization for hypertension, type II diabetes mellitus, and chronic kidney disease.

Supplementary table S6. Sensitivity analyses for association between pre-existing cardiovascular disease and the rate of receipt of fluoropyrimidines.

**Analysis**	**Estimates in HR/OR (95% CI)**
Extended analysis until end of study*	0.73 (0.70-0.75)
Hospital adjusted†	0.72 (0.70-0.75)
Performance status of 3 excluded and performance status adjusted‡	0.78 (0.75-0.81)
Performance status of 3 or 4 excluded and performance status adjusted‡	0.78 (0.75-0.81)
Logistic regression model§	0.63 (0.60-0.66)

HR=hazard ratio, OR=odds ratio, CI=confidence interval.

*Extending follow-up of all patients until the October 2022 data cut, rather than 1-year after the cancer diagnosis.

†Inclusion of the diagnosis hospital as an additional covariate in the Cox model.

‡Including performance status in the model.

§Using a logistic regression to investigate the probability of receiving fluoropyrimidine treatment within one year, ignoring the timing of treatment.

Supplementary table S7. Patient baseline demographics, tumour characteristics and baseline cardiovascular risk factors of patients with missing performance status or performance status of 4 excluded.

	**All patients N=66,015**	**Fluoropyrimidine N=37,961**	**No fluoropyrimidine N=28,054**
**Age in years (median [IQR])**	71 [62, 79]	66 [58, 73]	78 [70, 84]
**Female (%)**	25,353 (38.4)	13,885 (36.6)	11,468 (40.9)
**Ethnicity (%)**			
White	61,173 (92.7)	35,124 (92.5)	26,049 (92.9)
Asian	1,149 (1.7)	737 (1.9)	412 (1.5)
Black	892 (1.4)	549 (1.4)	343 (1.2)
Mixed	222 (0.4)	138 (0.4)	84 (0.3)
Other	622 (1.1)	432 (1.1)	190 (0.7)
Unknown	1,957 (3.0)	981 (2.6)	976 (3.5)
**Index of Multiple deprivation quintile (%)**			
1 - most deprived	12,473 (18.9)	6,699 (17.7)	5,774 (20.6)
2	12,730 (19.3)	7,222 (19.0)	5,508 (19.6)
3	13,863 (21.3)	8,076 (21.3)	5,787 (20.6)
4	14,182 (21.5)	8,378 (22.1)	5,804 (20.7)
5 - least deprived	12,767 (19.3)	7,586 (20.0)	5,181 (18.5)
**Performance status (%)**			
0	29,661 (44.9)	22,449 (59.1)	7,212 (25.7)
1	21,042 (31.9)	12,355 (32.6)	8,687 (31.0)
2	9,636 (14.6)	2,705 (7.1)	6,931 (24.7)
3	5,676 (8.6)	452 (1.2)	5,224 (18.6)
**Cancer site (%)**			
Oesophageal	16,094 (24.4)	8,980 (23.7)	7,114 (25.4)

Gastric	8,550 (13.0)	4,062 (10.7)	4,488 (16.0)
Small intestine	455 (0.7)	224 (0.6)	231 (0.8)
Colorectal	40,916 (62.0)	24,695 (65.1)	16,221 (57.8)
**Cancer histology (%)**			
Adenocarcinoma	55,129 (83.5)	33,648 (88.6)	21,481 (76.6)
Squamous cell carcinoma	6,187 (9.4)	3,548 (9.3)	2,639 (9.4)
Other	4,702 (7.1)	767 (2.0)	3,935 (14.0)
**Cancer stage (%)**			
II	4,889 (7.4)	2,571 (6.8)	2,318 (8.3)
III	33,757 (51.1)	20,912 (55.1)	12,845 (45.8)
IV	27,369 (41.5)	14,478 (38.1)	12,891 (46.0)
**Route to diagnosis (%)**			
Emergency presentation	13,302 (20.2)	5,223 (13.8)	8,079 (28.8)
GP referral	12,676 (19.2)	7,153 (18.8)	5,523 (19.7)
Inpatient elective	3,331 (5.1)	2,114 (5.6)	1,217 (4.3)
Other outpatient	3,415 (5.2)	1,896 (5.0)	1,519 (5.4)
Screening	3,145 (4.8)	2,558 (6.7)	587 (2.1)
Two-week wait	29,313 (44.4)	18,565 (48.9)	10,748 (36.7)
Unknown	833 (1.3)	452 (1.2)	381 (1.4)
**Previous hospitalization (%)**			
Arrhythmia	7,342 (11.1)	2,178 (5.7)	5,164 (18.4)
Previous Cardiac arrest	216 (0.3)	46 (0.1)	170 (0.6)
Chronic kidney disease	1,747 (2.7)	273 (0.7)	1,474 (5.3)
Coronary artery disease	10,180 (15.4)	3,729 (9.8)	6,451 (23.0)
Diabetes mellitus	4,233 (6.4)	1,324 (3.5)	2,909 (10.4)
Heart failure	3,276 (5.0)	724 (1.9)	2,552 (9.1)
Hypertension	12,283 (18.6)	4,124 (10.9)	8,159 (29.1)

Peripheral vascular disease	1,532 (2.3)	480 (1.3)	1,052 (3.8)
Stroke	1,735 (2.6)	452 (1.2)	1,283 (4.6)
Valvular heart disease	2,745 (4.2)	758 (2.0)	1,987 (7.1)
Venous thromboembolic disease	1,942 (2.9)	697 (1.8)	1,245 (4.4)
Any cardiovascular disease*	18,157 (27.5)	6,533 (17.2)	11,624 (41.4)

IQR=interquartile range.

***Any of the following diagnoses:** angina, myocardial infarction, revascularization procedures, percutaneous coronary interventions, coronary artery bypass graft surgery, deep venous thromboembolism, pulmonary embolism, heart failure and cardiomyopathy, valvular heart diseases, arrhythmia, peripheral vascular disease, sudden cardiac arrest, and stroke.
